# Influencing factors of significant breast edema in breast cancer patients undergoing breast-conserving surgery during radiotherapy with DIBH

**DOI:** 10.3389/fonc.2026.1819617

**Published:** 2026-04-24

**Authors:** Yong Sang, Xiaoye Su, Nan Hu, Jianan Wu, Lijuan Huang, Jing Jin, Qin Xiao

**Affiliations:** Department of Radiation Oncology, National Cancer Center/National Clinical Research Center for Cancer/Cancer Hospital & Shenzhen Hospital, Chinese Academy of Medical Sciences and Peking Union Medical College, Shenzhen, China

**Keywords:** breast edema, breast-conserving surgery, influencing factors, radiotherapy, significant breast edema

## Abstract

**Background and purpose:**

Breast-conserving surgery (BCS) followed by radiotherapy is the standard of care for most early-stage breast cancer. Although Breast edema (BE) following radiotherapy has been widely reported, current studies mainly focuses on factors influencing post-radiotherapy BE and its impact on quality of life. This study investigates the factors associated with significant Breast edema (SBE) during radiotherapy, which may lead to notable changes in target dose distribution and potentially necessitate replanning, aiming to provide guidance for clinical management.

**Methods:**

We retrospectively analyzed 144 patients who received radiotherapy after BCS at our institution from May 2024 to November 2025. All patients underwent deep inspiration breath-hold (DIBH) during simulation CT, cone beam computed tomography (CBCT) imaging, and treatment delivery. SBE during radiotherapy was defined as a breast dimensional increase of ≥5 mm in either the X or Z direction on at least one of three CBCT scans (obtained before the 1st, 6th, and 11th fractions), based on the institutional margin of 5 mm from clinical target volume (CTV) to planning target volume (PTV). Potential influencing factors included height, weight, body mass index (BMI), tumor location, age, TNM stage, chemotherapy, radiation energy, volumes of CTV, CTVboost, PTV, and PTVboost, as well as dosimetric parameters for PTVboost and PTV-PTVboost.

**Results:**

Univariate analysis showed that the volume of CTV, CTVboost, PTV and PTVboost were significant predictors of SBE during radiotherapy With odds ratios (OR) slightly above 1.000 indicating a positive association. Multivariate analysis identified PTVboost volume was the only independent factor significantly associated with increased risk of SBE (OR = 1.072, 95% CI: 1.010-1.137, p = 0.022). Receiver operating characteristic (ROC) curve analysis determined the optimal cutoff value of PTVboost volume for predicting SBE to be 107.5 cc, yielding a sensitivity of 0.800 and a specificity of 0.560.

**Conclusions:**

PTVboost volume was the only significant factor influencing the occurrence of SBE during radiotherapy. A larger PTVboost volume was associated with a higher probability of SBE, possibly related to surgical extent and volume receiving high-dose irradiation. Increased attention should be paid to SBE in BCS patients during radiotherapy due to its implications for treatment accuracy and patient quality of life. Proactive management of SBE is recommended in this setting.

## 1 Introduction

Breast cancer (BC) is the most common malignancy among women, accounting for approximately 25% of female cancers globally, with an estimated 2.3 million new cases in 2022 (1). Breast-conserving therapy (BCT), which consists of breast-conserving surgery (BCS) followed by adjuvant whole-breast radiotherapy, has become a standard treatment for early-stage breast cancer, offering oncological outcomes comparable to mastectomy (2). However, some patients develop breast edema (BE) in the treated breast following surgery and radiotherapy (3). Existing research on BE in BCS patients undergoing radiotherapy has primarily focus on identifying determinants of post-radiotherapy edema and developing interventions to alleviate it, aiming to improve patient quality of life (3–7). Few studies addressed the potential insufficiency of the CTV-to-PTV margin due to BE during radiotherapy, which may necessitate adaptive replanning. Meanwhile, the deep inspiration breath-hold (DIBH) technique has been widely adopted in breast radiotherapy due to its advantages over free breathing (FB) in sparing organs at risk (8–11).

To address this gap, this retrospective study enrolled BCS radiotherapy patients treated with DIBH-based radiotherapy. Through registration between simulation CT and pre-treatment CBCT images, we evaluated the occurrence of significant breast edema (SBE) during radiotherapy analyzed its risk factors to inform clinical practice.

## 2 Materials and methods

### 2.1 Patients population

We retrospectively analyzed 144 breast cancer patients who underwent radiotherapy after BCS between May 2024 and November 2025. This retrospective study was approved by Chinese Academy of Medical Sciences Cancer Hospital Shenzhen Hospital Ethics Committee (No. JS2023-17-1) and complied with the Declaration of Helsinki. Written informed consent was not required from all patients involved in the study. All patients were female: 77 with left-sided and 67 with right-sided tumors. According to clinical indications, 105 patients received 4–8 cycles of chemotherapy after surgery and before radiotherapy, while 39 proceeded directly to radiotherapy without chemotherapy. Baseline and clinical characteristics are summarized in **Table 1**.

**Table 1:** Baseline and clinical characteristics of BCS radiotherapy patients (n = 144, all female).

Characteristic	Value/subgroup	Number/mean ± SD or median (range)
Laterality	Left	77
	Right	67
T stage	T0	9
	T1	92
	T2	41
	T3	2
N stage	N0	132
	N1	12
M stage	M0	144
Chemotherapy	Yes	105
	N0	39
Beam energy	6 MV	103
	6 MV FFF	41
Age (years)		43.7 ± 9.1 (25-68)
Height (cm)		159.3 ± 5.7 (145-172)
Weight (kg)		59.0 ± 7.6 (42-83)
BMI		23.2 ± 2.9 (16.6-31.4)
CTV (cc)		405.0 ± 172.9 (99–1001)
CTVboost (cc)		67.7 ± 33.0 (20–189)
PTV (cc)		565.2 ± 196.3 (203–1194)
PTVboost (cc)		118.5 ± 49.0 (38–285)
PTVboost Dmean (cGy)		5120.8 ± 25.2 (5046–5182)
PTVboost Dmax (cGy)		5368.5 ± 64.2 (5228–5574)
PTVboost V107 (%)		0.91 ± 1.96 (0–13.02)
PTV-PTVboost Dmean (cGy)		4557.3 ± 25.6 (4490–4642)
PTV-PTVboost Dmax (cGy)		5256.6 ± 60.5 (5122–5497)
PTV-PTVboost V107 (%)		17.82 ± 7.04 (6.88–41.52)

FFF, flattening filter free; BMI, body mass index, cc, cubic centimeter; Dmean, mean dose; Dmax, maximum dose; V107, volume receiving ≥107% of prescription dose.

### 2.2 Treatment characteristics and radiotherapy planning

*Simulation and Target Delineation:* All patient underwent CT simulation in the supine position with both arms raised above the head. Simulation was performed using a GE Discovery RT590 scanner (GE Healthcare) with DIBH technique was employed during simulation to achieve reproducible breath-hold at deep inspiration. Based on the recommendations of relevant guidelines, our institution requires the use of DIBH technology as much as possible for left-sided breast cancer patients (12, 13). For right-sided breast cancer patients, the use of DIBH is recommended based on the protection of the lungs and liver (10, 11), and this cohort includes all patients who underwent DIBH, regardless of side.

The clinical target volume (CTV) encompassed the whole ipsilateral breast parenchyma and the fascia of the pectoralis major muscle. The tumor volume for boost (CTVboost) was defined as the surgical cavity, including any seroma, surgical clips, and post-operative architectural distortion. Standardized margin expansions were applied: the planning target volume (PTV) was generated by expanding the CTV by 1.0 cm superiorly-inferiorly and 0.5 cm in all other directions. The PTVboost was created by expanding the CTVboost uniformly by 1.0-1.5 cm in all directions, followed by cropping 5 mm from the skin surface (14).

Organs at risk (OARs), including the heart, bilateral lungs, contralateral breast, and spinal cord, were contoured according to established guidelines (15).

**Figure 1:**
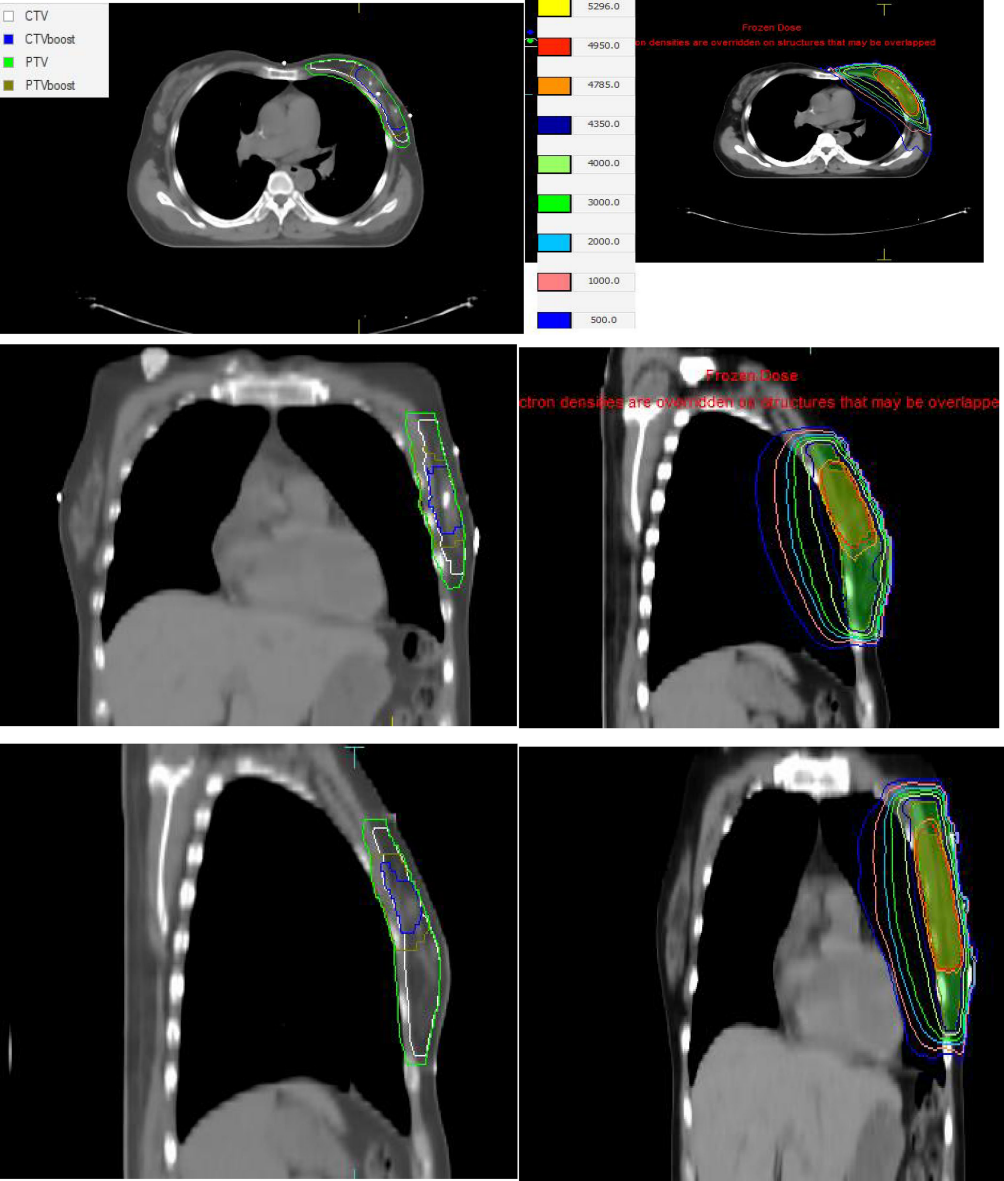
**(A)** The targets of CTV, CTVboost, PTV, PTVboost for a patient. **(B)** The dose distribution map for a patient.

Dosimetry and Treatment Delivery: All patients were prescribed a hypofractionated regimen: 43.5 Gy in 15 fractions to the whole breast (PTV), followed by a simultaneous integrated boost (SIB) of 49.5 Gy in 15 fractions to the tumor bed (PTVboost). This regimen is biologically equivalent to 50 Gy in 25 fractions plus a 10 Gy in 5 fractions boost, assuming an α/β ratio of 4.0 Gy for breast cancer tumor control (**Figure 1**) (14, 16).

Treatment plans were generated using the Monaco 6.0 treatment planning system (TPS) (Elekta AB, Stockholm, Sweden). For most patients, a hybrid technique combining two tangential 6 MV photon fields with a partial-arc volumetric modulated arc therapy (VMAT) segment was utilized. If OAR constraints were exceeded (ipsilateral lung V20Gy<25%, V5Gy<55%, mean dose<14 Gy; heart mean dose<5 Gy; contralateral breast mean dose<5 Gy), a full-arc VMAT plan using 6 MV flattening filter free (FFF) beams was employed (17). An autoflash margin of 2.0 cm was applied to all plans to account for superficial tissue (18). Dose calculation was performed using the X-ray Voxel Monte Carlo (XVMC) algorithm with a 3 mm calculation grid and 1% per plan statistical uncertainty (19).

All treatments were delivered on an Elekta Infinity linear accelerator equipped with an Agility multileaf collimator. Daily image guidance was performed using CBCT acquired under DIBH conditions prior to each fraction. Offline reviews ensured consistent breath-hold levels.

### 2.3 Simulation CT and CBCT analysis

**Figure 2:**
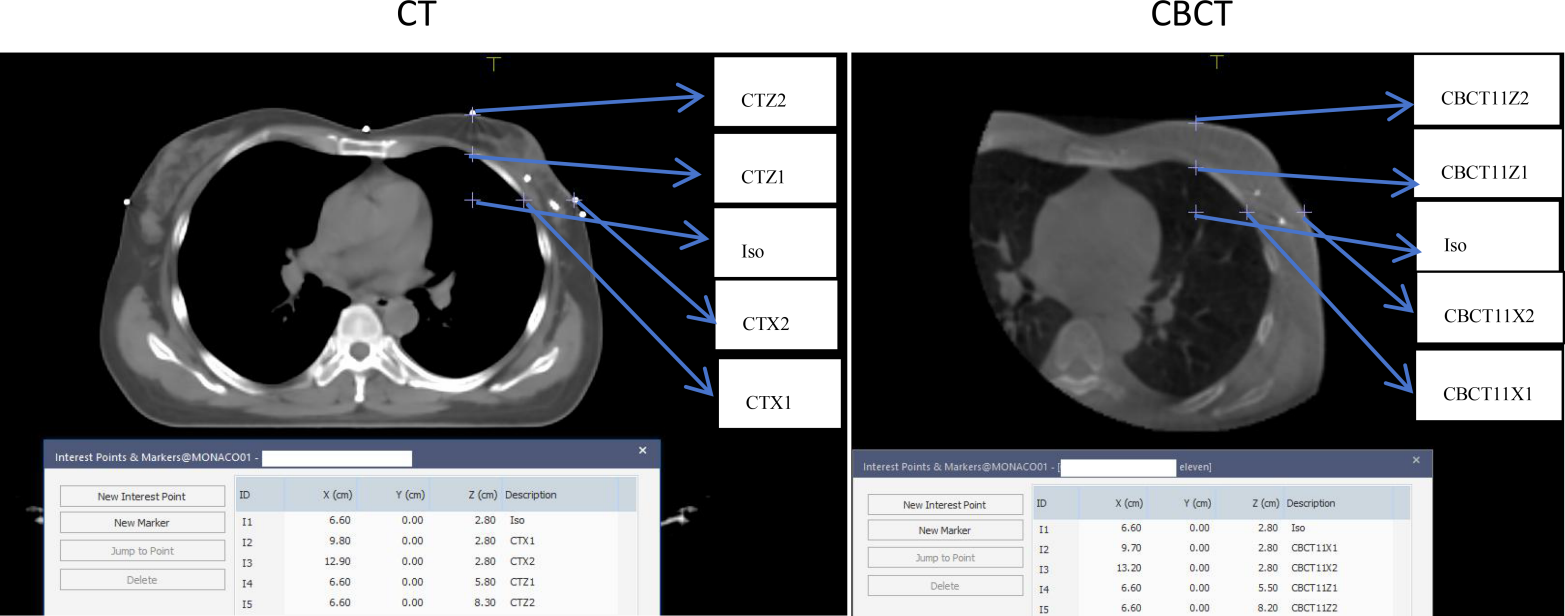
Breast dimension measurements diagram for CT and CBCT.


To quantify SBE, breast dimensions in the X and Z directions were measured at the isocenter on simulation CT and on three CBCT scans (before fractions 1, 6, and 11). The isocenter (ISO) was defined at the center of PTV in the Y direction, with X located medially/superiorly relative to the nipple and Z near the mid-axillary line (**Figure 2**). On CT, points CTX1 and CTX2 represented the inner and outer breast boundaries along the X-direction line through ISO; CTZ1 and CTZ2 were similarly defined for the Z direction. Dimensions were calculated as:

$${\text{X}}_{\text{CT}}=|{\text{X}}_{\text{CTX}1}-\;{\text{X}}_{\text{CTX}2}|$$

$${\text{Z}}_{\text{CT}}=|{\text{Z}}_{\text{CTZ}1}-\;{\text{Z}}_{\text{CTZ}2}|$$

Corresponding measurements were performed on registered CBCT images (e.g., X_CBCT1_ = |X_CBCT1X1_- X_CBCT1X2_|),. Changes relative to baseline were computed as ΔX_CBCT1_ = X_CBCT1_ - X_CT_ andΔZ_CBCT1_ = Z_CBCT1_ - Z_CT_ as shown in **Figure 2**. SBE was defined as ΔX orΔZ ≥ 5 mm on any of the threes CBCT scans, based on the institutional CTV-to-PTV margin of 5 mm in these direction. || represents the absolute value.

### 2.4 Potential influencing factors

The analyzed factors included: tumor location, height, weight, body mass index (BMI), age, TNM stage, chemotherapy, radiation energy, volumes of CTV, CTVboost, CTVboost/CTV, PTV, PTVboost and PTVboost/PTV, as well as dosimetric parameters for PTVboost [mean dose (D_m_), maximum dose (D_max_), volume percentage of the target region exceeding 107% of the prescription dose (V_107_)] and PTV-PTVboost (D_m_, D_max_, V_107_). PTV-PTVboost refers to the volume of PTV minus the volume of PTVboost.

### 2.5 Statistical analysis

Patients were classified into SBE and non-SBE groups. Categorical variables were compared using chi-square test, continuous variables with independent t-tests. Univariate binary logistic regression was performed for all potential factors. Variables with p ≤ 0.05 in univariate analysis were entered into multivariate logistic regression to identify independent predictors. For continuous independent receiver operating characteristic (ROC) curves were plotted to determine optimal cutoff values. Analyses were performed using SPSS (v26.0), with p< 0.05 considered statistically significant.

## 3 Results

### 3.1 Incidence of significant breast edema

**Figure 3:**
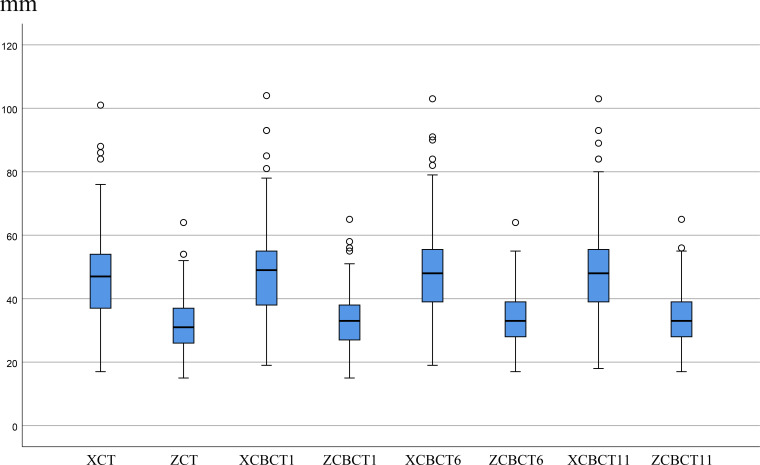
Box plots of X and Z dimensions for CT and CBCT. mm, millimeter; XCT, X_CT_, the X-direction breast dimension at the isocenter on CT; ZCT, Z_CT_, the Z-direction breast dimension at the isocenter on CT; XCBCT1, X_CBCT1_, the X-direction breast dimension at the isocenter on CBCT of the first treatment; ZCBCT1, Z_CBCT1_, the Z-direction breast dimension at the isocenter on CBCT of the first treatment; XCBCT6, X_CBCT6_, the X-direction breast dimension at the isocenter on CBCT of the sixth treatment; ZCBCT6, Z_CBCT6_, the Z-direction breast dimension at the isocenter on CBCT of the six treatment; XCBCT11, Х_CBT11_, the X-direction breast dimension at the isocenter on CBCT of the eleventh treatment; ZCBCT11, Z_CBCT11_, the Z-direction breast dimension at the isocenter on CBCT of the eleventh treatment.

**Figure 4:**
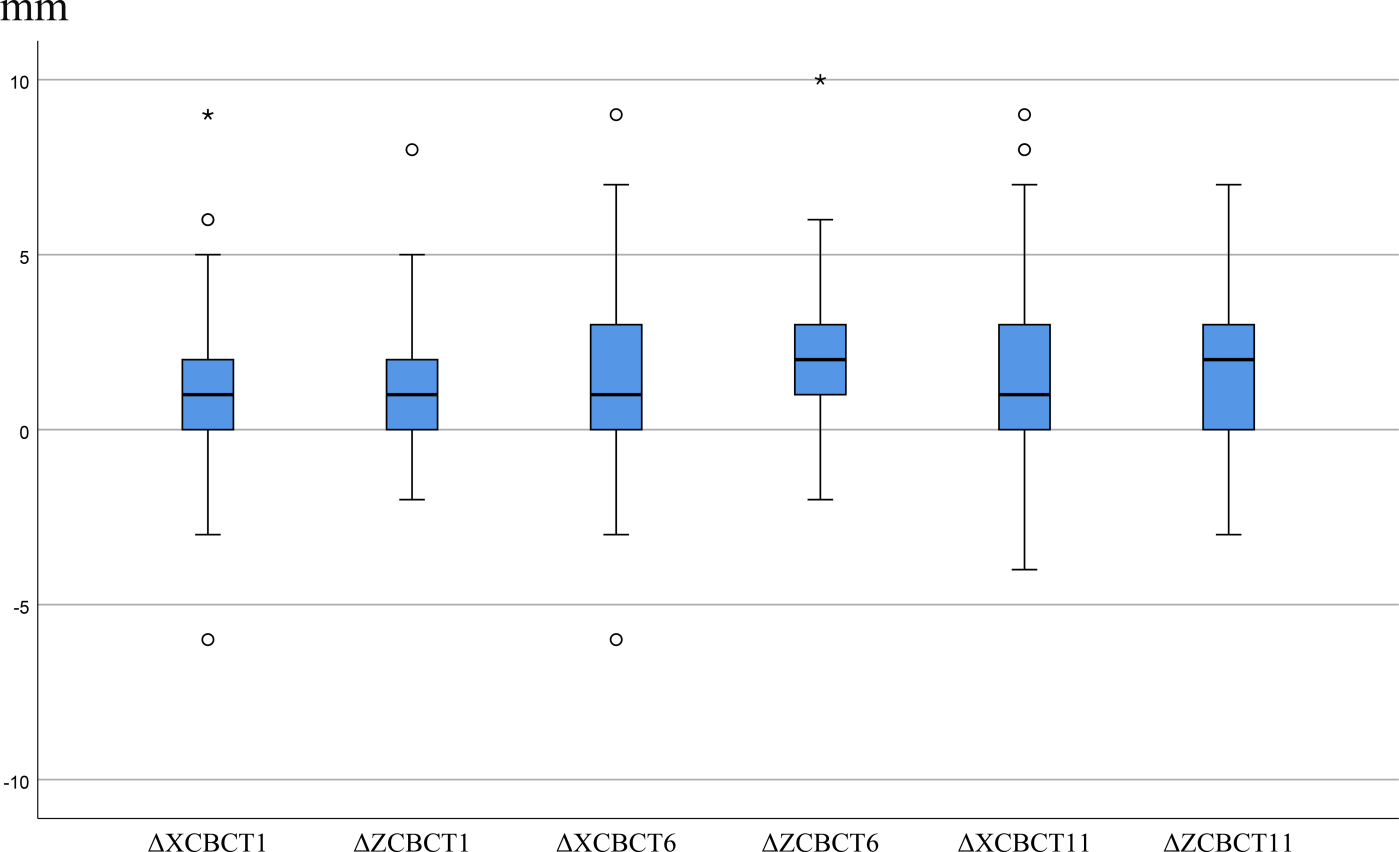
Box plots of ΔX and ΔZ dimensions for CBCT. mm, millimeter; ΔХСВСТІ, ΔХ_СBCTI_, the breast dimension difference in the X-direction at the isocenter between the first treatment CBCT and the simulation CT; ΔZCBCT1, ΔZ_CBCTI_, the breast dimension difference in the Z-direction at the isocenter between the first treatment CBCT and the simulation CT; ΔХСВСТ6, ΔХ_СBCT6_, the breast dimension difference in the X-direction at the isocenter between the sixth treatment CBCT and the simulation CT; ΔZCBCT6, ΔZ_CBCT6_, the chest breast dimension in the Z-direction at the isocenter between the sixth treatment CBCT and the simulation CT; ΔXCBCT11, ΔX_CBCT1_, the breast dimension difference in the X-direction at the isocenter between the eleventh treatment CBCT and the simulation CT; ΔZCBCT11, ΔZ_CBCT11_, the breast dimension difference in the Z-direction at the isocenter between the eleventh treatment CBCT and the simulation CT. The asterisk (*) represents extreme outliers. These values are more extreme, lying beyond 3 times the interquartile range (IQR) from the box.

Breast dimensions increased during radiotherapy, with peak means at fractions 6 and 11. Changes in the Z-direction were generally larger than in the X-direction. Based on the defined threshold, SBE was observed in 8 patients (8.0%) at fraction 1, 23 (16.0%) at fraction 6, and 25 (17.4%) at fraction 11. Cumulatively, 35 patients (24.3%) experienced SBE in at least one direction during radiotherapy. Maximum edema reached 10 mm (**Figures 3**, **4**).

### 3.2 Influencing factor analysis

Univariate analysis showed that CTV, CTVboost, PTV, and PTVboost volumes were significantly larger in the SBE group (all p< 0.05). No significant differences were found for tumor location, age, T/N stage, chemotherapy, energy, or dosimetric parameters (**Table 2**).

**Table 2:** Comparisons between patients with and without SBE during radiotherapy.

Factors	SBE (n=35)	Non-SBE (n=109)	P value
Age (years)	45.2 ± 9.9	43.2 ± 8.9	0.268
Height (cm)	158.8 ± 6.6	159.5 ± 5.4	0.518
Weight (kg)	60.4 ± 7.2	58.5 ± 7.8	0.202
BMI	24.0 ± 2.8	23.0 ± 2.9	0.078
CTV (cc)	466.8 ± 180.5	385.1 ± 163.4	**0.013**
CTVboost (cc)	80.8 ± 37.7	63.4 ± 30.3	**0.006**
CTVboost/CTV	0.178 ± 0.054	0.173 ± 0.059	0.685
PTV (cc)	638.5 ± 203.3	541.6 ± 189.0	**0.011**
PTVboost (cc)	141.1 ± 54.8	111.2 ± 45.0	**0.002**
PTVboost/PTV	0.225 ± 0.060	0.210 ± 0.057	0.204
PTVboost D_m_ (cGy)	5123 ± 25	5120 ± 25	0.490
PTVboost D_max_ (cGy)	5360 ± 57	5371 ± 66	0.363
PTVboost V_107_ (%)	0.65 ± 1.20	0.99 ± 2.15	0.374
PTV-PTVboost D_m_ (cGy)	4562 ± 24	4556 ± 26	0.161
PTV-PTVboost D_max_ (cGy)	5252 ± 54	5258 ± 62	0.584
PTV-PTVboost V_107_ (%)	18.4 ± 6.7	17.6 ± 7.2	0.576
Location			0.781
Left	18	59	
right	17	50	
pT			0.662
0	2	9	
1	25	67	
2	8	33	
3	0	2	
pN			0.953
0	32	100	
1	3	9	
chemotherapy			0.270
Yes	23	82	
No	12	27	
Energy			0.088
6MV	29	74	
6MV FFF	6	35	

SBE, significant breast edema during radiotherapy; Non-SBE, no significant breast edema during radiotherapy; cc, cubic centimetre; CTVboost/CTV, the ratio of CTVboost volume to CTV volume; PTVboost/PTV, the ratio of PTVboost volume to PTV volume; Dmean, mean dose; Dmax, maximum dose; V107, volume percentage of the target region exceeding 107% of the prescription dose; PTV-PTVboost, the volume of PTV minus PTVboost.

The bold values indicate that the P value is less than 0.05.

Univariate logistic regression conformed positive associations for CTV (OR = 1.003, 95% CI: 1.000-1.005, p = 0.017), CTVboost (OR = 1.015, 95% CI: 1.004-1.026, p = 0.009), PTV (OR = 1.002, 95% CI: 1.001-1.004, p = 0.013), and PTVboost (OR = 1.012, 95% CI: 1.004-1.020, p = 0.003).

Multivariable logistic regression analysis revealed that only PTVboost volume remained an independent risk factor for edema (OR = 1.072, 95% CI: 1.010–1.137, p = 0.022). It is noteworthy that the association of CTVboost reversed direction and was of borderline significance (OR = 0.916, 95% CI: 0.840–0.999, p = 0.048). Due to the extremely high correlation between PTVboost and CTVboost (Pearson’s r = 0.981), which indicates substantial multicollinearity, the coefficient estimates for these two variables in the multivariable model should be interpreted with caution. The reversal of the OR for CTVboost is likely a statistical artifact of this collinearity, rather than a true protective effect. Therefore, this result should be interpreted as indicating that CTVboost did not demonstrate an independent positive effect after adjusting for the influence of PTVboost, rather than suggesting a protective effect. Neither PTV nor CTV reached statistical significance in the multivariable model (P≥0.05). These findings suggest that the volume effect of PTVboost dominates in the multivariable context. Based on this, subsequent analysis will focus on the PTVboost volume through ROC curve analysis to evaluate its predictive value.

**Figure 5:**
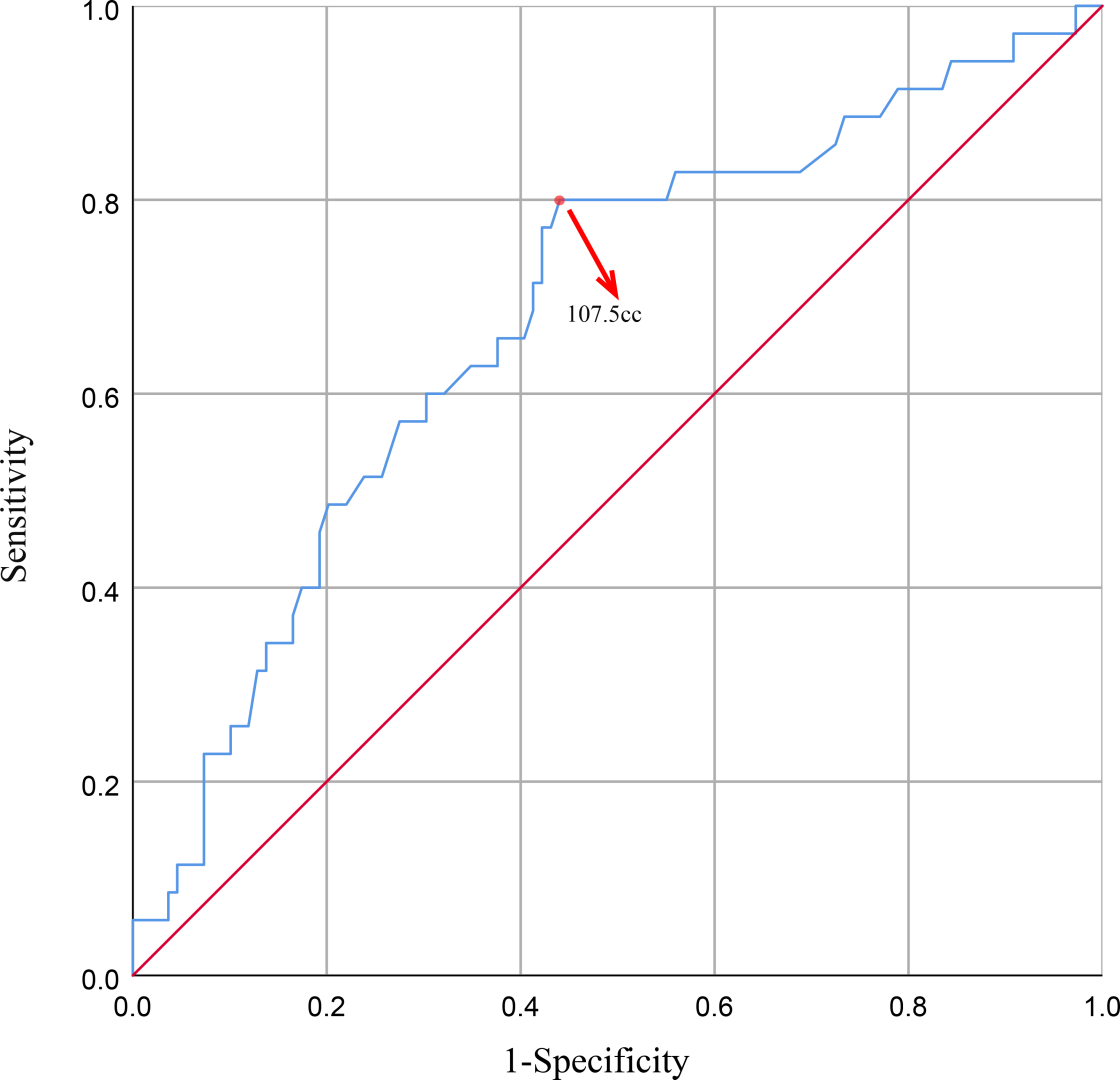
The ROC curve for PTVboost.

ROC analysis for PTVboost volume yielded an AUC of 0.682 (95% CI: 0.579-0.784, p = 0.001). The optimal cutoff was 107.5 cc [sensitivity 0.800, specificity 0.560 (**Figure 5**)].

Based on the optimal cutoff value of 107.5 cc, patients were stratified into high-risk (PTVboost > 107.5 cc, n = 76) and low-risk (PTVboost ≤ 107.5 cc, n = 68) groups. The positive predictive value (PPV) for SBE in the high-risk group was 36.8% (28/76), and the negative predictive value (NPV) was 89.7% (61/68). Patients with PTVboost > 107.5 cc had a significantly higher incidence of SBE compared to those with PTVboost ≤ 107.5 cc (36.8% vs. 10.3%, p< 0.001), with a relative risk of 3.58 (95% CI: 1.67-7.68). The high NPV suggests that patients with PTVboost volumes below this threshold are unlikely to develop SBE during radiotherapy and may not require intensive monitoring.

## 4 Discussion

BE following BCS and radiotherapy represents a well-documented clinical concern in breast cancer management. While previous studies have predominantly examined post-radiotherapy edema and its impact on long-term quality of life (3–7), factors associated with BE during radiotherapy remain largely unexplored. Notably, SBE occurring during treatment may not only affect patients’ quality of life but also compromise dose-delivery accuracy and treatment efficacy due to anatomical deformation. Moreover, prior analyses of post-radiotherapy BE have often been limited to binary assessments of radiotherapy exposure, lacking integration of specific dosimetric parameters-such as target volumes and dose distributions-into risk-factor modeling (3–7). To our knowledge, this is the first study to investigate dosimetric and clinical predictors of SBE during radiotherapy using CBCT-based dimensional assessment.

In this cohort, 24.3% of patients developed SBE during treatment—a proportion that differs from some reported incidences of post-radiotherapy breast edema. For instance, Cornacchia et al. (4) reported a 5.8% incidence of breast edema in a cohort of 485 patients who received whole-breast irradiation after BCS. Several factors may account for this discrepancy. First, our definition of SBE was based on a≥5 mm dimensional increase in the X or Z direction at the isocenter, corresponding to our institutional CTV-to-PTV margin; this dosimetrically oriented threshold differs from the symptom-or device-based definitions used in many earlier studies. Second, the timing of assessment likely plays a critical role: while previous studies typically evaluated edema after radiotherapy completion or at the end of combined modality treatment, we focused on edema during the radiotherapy course, which may capture transient edema that resolves by follow-up. Third, methodological differences are notable: some prior studies relied on patient-reported outcomes or remote assessments (e.g., telephone surveys during the COVID-19 pandemic) and encountered challenges in obtaining objective measurements (e.g., tissue dielectric constant or skin stiffness assessments) for all suspected cases, potentially leading to under-reporting. Finally, our cohort exclusively comprised patients treated with deep-inspiration breath-hold (DIBH) technique, whereas earlier reports seldom specified radiotherapy techniques in detail. It is worth noting that the reported incidence of breast edema after BCS plus radiotherapy varies widely in the literature, ranging from 0% to 90.4% (3), reflecting substantial heterogeneity in assessment methods and definitions of BE/SBE.

Consistent with part of the literature (4), we found no significant association between SBE and laterality, age, TN stage (all patients were M0), prior chemotherapy, or planned energy (6 MV vs. 6 MV FFF). The role of chemotherapy remains controversial: some studies have reported higher degrees of BE in patients receiving chemotherapy (20), whereas others suggested a protective effect against acute BE (21). Similarly, the relationship between age and BE has been inconsistently reported (4, 20, 21), likely due to variations in edema definitions and measurement approaches across studies.

Notably, high-dose volumetric parameters (V107, Dmax) were not associated with SBE in our analysis. This may be explained by our institutional planning protocols, which strictly limit the PTVboost V107 to<1% for most conventional breast-conserving cases (unless organ-at-risk constraints necessitate relaxation). Consequently, the cohort exhibited very low high-dose volumes, with V110 being 0% in the majority of patients—hence our selection of V107 as the high-dose metric. Another plausible explanation is that chest-wall deformation during treatment could alter the actual high-dose distribution relative to the planned dose, thereby decoupling the planned V107 from the delivered dose to the swollen anatomy.

Univariate analysis identified CTV, CTVboost, PTV, and PTVboost volumes as significant predictors of SBE. However, in the multivariate model, only PTVboost volume remained independently significant (OR = 1.072, p = 0.022), while CTVboost exhibited a direction-reversed, borderline-significant association (OR = 0.916, p = 0.048) likely due to high collinearity with PTVboost (r = 0.981). The increase in the OR for PTVboost from univariate (1.012) to multivariable analysis (1.072) can be attributed to the redistribution of shared variance between PTVboost and the highly collinear CTVboost. After adjusting for CTVboost in the model, the independent effect of PTVboost became more clearly delineated. The strong correlations among these volumetric measures (PTVboost vs. PTV r = 0.712; PTVboost vs. CTV r = 0.711) explain why the multivariate model isolated PTVboost as the dominant predictor. This finding aligns with the clinical rationale that a larger boost volume—reflecting both the extent of surgical resection and the tissue receiving high-dose per-fraction irradiation (3.3 Gy/fraction)—would reasonably increase the probability of acute, symptomatic edema.

From a mechanistic perspective, the independent predictive value of PTVboost volume can be attributed to several factors. First, the higher dose per fraction delivered to the PTVboost (3.3 Gy) compared to the whole breast (2.9 Gy) likely induces a more pronounced acute inflammatory response, including endothelial damage and increased capillary permeability, promoting fluid extravasation specifically within the high-dose region. Second, the PTVboost encompasses the surgical cavity, where lymphatic vessels have been disrupted by surgery, impairing lymphatic drainage. The subsequent high-dose irradiation exacerbates this pre-existing lymphatic insufficiency, leading to fluid stasis. Third, the PTVboost includes a margin of surrounding normal breast tissue; a larger PTVboost volume therefore indicates a greater volume of normal tissue exposed to this biologically effective dose, amplifying the overall acute inflammatory response. These combined mechanisms—high dose per fraction effect, surgical lymphatic disruption, and volume effect—provide biological plausibility for PTVboost volume as the dominant predictor of SBE.

The high negative predictive value (89.7%) of the 107.5 cc threshold, as demonstrated in our results, suggests that this cutoff could serve as a reliable screening tool to identify low-risk patients who may not require intensive monitoring during radiotherapy. Conversely, patients with PTVboost volumes above this threshold have a 3.6-fold increased risk of SBE and may benefit from closer observation or adaptive replanning.

Our results carry direct clinical implications. The observed 24.3% incidence of SBE during DIBH-based radiotherapy suggests that a substantial proportion of patients may experience anatomical changes sufficient to perturb dose delivery. For such patients, verification of delivered dose accuracy and consideration of adaptive replanning may be warranted. Furthermore, significant chest-wall deformation may necessitate re-acquisition of the DIBH reference surface to ensure reproducible positioning. Although our institutional margin is 5 mm in the X and Z directions, practical trimming of the PTV/PTVboost from the subcutaneous tissue reduces the effective margin; the registration accuracy of CBCT, while significantly reducing positional discrepancies between planned and actual treatment, still carries a margin of error of approximately 1 mm (22), thus, a 5-mm threshold for defining SBE is both dosimetrically relevant and institutionally justified.

This study is the first to establish the association between PTVboost volume and SBE under DIBH technique. However, the patterns and incidence of SBE may differ under free-breathing (FB) conditions, which serves as a standard control. To clarify the impact of different respiratory management techniques on the risk of SBE, a direct comparative study incorporating an FB patient cohort is currently underway at our institution.

Several limitations of this study should be acknowledged. First, dimensional changes were assessed only at the isocenter using the planning CT and three CBCT scans. Given the regional heterogeneity of edema, this approach may underestimate the incidence of SBE, suggesting that the actual rate during radiotherapy could be higher. Future investigations should employ more comprehensive three-dimensional volumetric change analysis of the breast, for instance by delineating the CTV on serial CBCTs. Combining volumetric changes with unidimensional measurements at the isocenter would provide a more holistic and accurate assessment of edema. Second, the 5-mm threshold for defining SBE is institution-specific and may not generalize to centers using different margin strategies. Additionally, whether a ≥5 mm breast deformation actually compromises dose delivery remains to be investigated in future studies. Third, our definition of SBE includes both true physiological edema and setup-related breast deformation, which could introduce measurement noise. Fourth, although all CBCT-CT registrations were verified by an experienced physician to ensure alignment accuracy, inherent uncertainties in registration could still influence the results. Fifth, as a retrospective study, we were unable to incorporate certain clinically relevant patient-reported outcomes, such as quality-of-life assessments and cosmetic evaluations following treatment. Prospective studies are therefore planned to systematically evaluate the impact of SBE on patients’ quality of life and satisfaction from their perspective, which will add an essential humanistic dimension to our findings. Sixth, as a single-center study with a modest sample size of 144 patients, our findings require validation in larger, multi-institutional cohorts. Finally, we did not incorporate radiomic features from simulation CT, which represents a promising avenue for future predictive modeling.

Additionally, regarding the clinical significance of defining SBE based on a ≥5 mm threshold, further validation is warranted. Although this cutoff is derived from our institutional CTV-to-PTV margin, it remains unverified whether this magnitude of breast deformation actually compromises the delivered dose distribution to the target volume or organs at risks (OARs). To address this, future studies at our institution will utilize CBCT-derived synthetic CT (sCT) techniques (23) to perform dose reconstruction. This approach will enable a direct evaluation of whether SBE leads to target underdosing or OAR overdosing, thereby substantiating the dosimetric relevance of the defined threshold.

## 5 Conclusion

PTVboost volume was the only independent factor associated with SBE during radiotherapy in BCS patients treated with DIBH. A larger PTVboost volume increased the probability of SBE, likely due to greater surgical and high-dose irradiation volumes. Clinicians should be aware of SBE during radiotherapy due to its potential impact on treatment accuracy and quality of life. Proactive monitoring and management are recommended for at-risk patients. As a single-center study with a limited sample size (n=144), these findings warrant validation in larger, multi-institutional cohorts.

## Data Availability

The raw data supporting the conclusions of this article will be made available by the authors, without undue reservation.
